# Recombinant Human Bone Morphogenetic Protein-2 (rhBMP-2) for the Treatment of Nonunion of the Femur in Children and Adolescents: A Retrospective Analysis

**DOI:** 10.1155/2017/3046842

**Published:** 2017-03-02

**Authors:** Tim N. Hissnauer, Norbert Stiel, Kornelia Babin, Martin Rupprecht, Karsten Ridderbusch, Johannes M. Rueger, Ralf Stuecker, Alexander S. Spiro

**Affiliations:** ^1^Department of Trauma, Hand, and Reconstructive Surgery, University Medical Center Hamburg-Eppendorf, Hamburg, Germany; ^2^Department of Pediatric Orthopaedic Surgery, Children's Hospital Hamburg-Altona, Hamburg, Germany; ^3^Department of Orthopaedics, University Medical Center Hamburg-Eppendorf, Hamburg, Germany

## Abstract

*Background*. The aim of this study was to examine clinical and radiographic healing after rhBMP-2 application in children and adolescents presenting with nonunion of the femur and to investigate the safety of rhBMP-2 use in these cases.* Materials and Methods*. We reviewed the medical records of five patients with a mean age of 11 years (5.4 to 16.2) with nonunion of the femur who were treated with rhBMP-2 and internal fixation using a locking plate at a single institution. Particular attention was paid to identify all adverse events that may be due to rhBMP-2 use.* Results*. Union occurred in four of five patients at a mean of 12.1 months (7.9 to 18.9). The locking plates were removed after a mean of 16 months (11 to 23). One patient had nonunion due to deep infection. After a mean follow-up of 62.5 months (17 to 100), union was still evident in all four patients and they were fully weight-bearing without pain.* Discussion*. In this retrospective study, rhBMP-2 combined with a locking plate has been used successfully to treat children and adolescents with nonunion of the femur in four of five cases. One major complication was thought to be possibly related to its use.

## 1. Introduction

The treatment of nonunion of long bones often requires a multimodal therapy including numerous operations. Surgical methods comprise resection of the nonvascular tissue at the nonunion site, external or internal fixation, and the use of autogenous bone graft or bone graft substitutes [1–4]. Due to their important role in bone formation and fracture healing by being involved in the cascade of cellular events of tissue formation and regeneration, bone morphogenetic proteins (BMPs) have gained increased attention in recent years [5–11]. Several clinical studies have demonstrated that recombinant human BMP-2 and rhBMP-7 improve the healing of bone defects in adults, since the Food and Drug Administration (FDA) approved their clinical use for specific indications [12, 13]. Although there is an off-label use of the recombinant form of both BMPs acknowledged by the FDA for the treatment of children in case of informed consent for minors and their parents about potential risks, there is a lack of studies reporting on BMP application in this patient population [14, 15]. Few studies demonstrated promising results of the use of rhBMP-2 and rhBMP-7 in children, especially for spine fusion and for the treatment of congenital pseudarthrosis of the tibia [14, 16–19]. Application of rhBMP-2 for the treatment of nonunion of the femur was reported in only one previous study (2 cases), focusing on complications associated with BMP use at different sites in 81 children [15]. The aim of this retrospective study was (1) to examine clinical and radiographic healing after rhBMP-2 application in children and adolescents presenting with nonunion of the femur and (2) to verify the safety of rhBMP-2 use in these cases.

## 2. Materials and Methods

We reviewed the medical records and radiographs of all patients with nonunion of the femur who had been treated with rhBMP-2 and internal fixation using a locking plate at the Pediatric Orthopaedic Department of Children's Hospital Hamburg-Altona, Hamburg, Germany (Table 1). Five patients were included in this study. Their mean age at operation was 11 years (5.4 to 16.2). The most frequent underlying disorder was infantile cerebral palsy associated with coxa antetorta in two patients (cases 1 and 4) and hip dislocation in another patient (case 3). Both of the patients with coxa antetorta underwent femoral derotation osteotomy with use of a locking plate. Femoral shortening osteotomy with locking plate fixation was combined with Pemberton osteotomy to treat the patient with hip dislocation. They all had conservative management of nonunion of the femur after surgery with screw breakage in two cases (cases 1 and 3) before rhBMP-2 was used. Operative treatment of femoral nonunion had failed in two patients prior to rhBMP-2 application in this series (cases 2 and 5). One of these had congenital femoral deficiency with leg length inequality (case 2). Femoral derotation osteotomy and lengthening with use of an Ilizarov device was performed first. Due to persistent femoral nonunion, this patient had undergone four operations prior to rhBMP-2 application including two pin revisions, exchange from Ilizarov device to monorail external fixator, and implantation of a locking plate, with plate breakage. In the other patient, femoral lengthening with free vascularized fibular grafting was used to treat leg length inequality after septic coxitis with osteomyelitis of the proximal femur and previous femoral head reconstruction (case 5). This patient developed a nonunion between the fibular graft and the proximal femur after surgery; he underwent three operations with replacement of the inserted K-wire to improve fixation of the fibular graft to the proximal femur (2 procedures) and removal of the K-wire before rhBMP-2 was applied.

The mean time interval between the primary operation, leading to nonunion of the femur, and rhBMP-2 application was 11.3 months. Informed consent for the use of rhBMP-2 was obtained from the parents in each case.

### 2.1. Surgical Treatment

With the patient supine on a radiolucent table, the nonvascular tissue at the nonunion site was excised carefully and a locking plate was inserted in all patients. In addition to rhBMP-2 application, autogenous bone grafting was used in two patients, and an external fixator was applied in one case. The wound was irrigated and appropriate attention was paid to haemostasis, before rhBMP-2 (InductOs; Medtronic Sofamor Danek, Münster, Germany), prepared with collagen sponges, was placed around the nonunion site at the end of the operation. The soft tissue was closed using interrupted sutures (Table 2).

### 2.2. Postoperative Care

Follow-up examinations were performed at approximately six-week intervals until radiological healing of the femoral nonunion appeared and every six to twenty-four months thereafter. Radiological healing was defined as evidence of bony bridging across the femoral cortical defect at the nonunion site on the anteroposterior and lateral view.

Particular attention was paid to identifying all adverse events that may be due to rhBMP-2 use, including allergic reactions, systemic toxicity, local inflammatory reactions, excessive wound swelling, hematoma, compartment syndrome, heterotopic ossification, infection, carcinogenicity, and excessive bone growth.

## 3. Results

**Figure 1:**
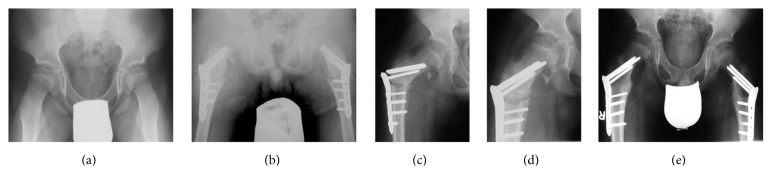
((a) and (b)) Anteroposterior preoperative (a) and postoperative (b) radiographs of a 10-year-old boy with infantile cerebral palsy with coxa antetorta who underwent femoral derotation osteotomy with use of a locking plate on both sides. (c) Anteroposterior radiograph of the same patient demonstrates persistent femoral nonunion with screw breakage of two femoral neck screws on the right side. (d) Anteroposterior radiograph two days after surgical revision with removal of the locking plate, resection of the femoral nonunion, application of rhBMP-2, and insertion of a new locking plate. (e) Anteroposterior radiograph 7.9 months after surgical intervention with use of rhBMP-2 demonstrating complete radiological healing which was still evident at the most recent follow-up, 60 months after rhBMP-2, was applied.

Intraoperative complications were not encountered. The average hospital stay was 7.6 days (4 to 10). Patients were not allowed to bear weight after rhBMP-2 application until radiographic healing of the nonunion was evident. Three patients mainly walked with crutches and used a wheelchair only for longer distances, while two patients were mobilized in a wheelchair after surgery. Radiological and clinical healing of the femoral nonunion occurred in four of five patients at a mean of 12.1 months (7.9 to 18.9) after rhBMP-2 use (Figure 1). The locking plates were removed after a mean of 16 months (11 to 23). Follow-up was a mean of 51.2 months (6 to 100). At the most recent follow-up, union was evident in four of five patients, and they were fully weight-bearing without pain (Table 3).

Complications that were thought possibly to be related to the use of rhBMP-2 included postoperative swelling and overheating of the wound in two cases, with spontaneous regression of the symptoms in one of the cases (case 4). The other patient (case 3) underwent surgical revision with wound debridement and application of antibiotic bead chains two months after rhBMP-2 application. Microbiological analysis revealed* Staphylococcus aureus*. Antibiotic therapy with Cefuroxime was started. Due to persistent signs of infection and plate loosening, this patient had revision surgery 2.5 months later with debridement, exchange of the antibiotic bead chains, and removal of the locking plate.* Staphylococcus aureus* was isolated from the cultures and antibiotic treatment with Cefazolin was initiated according to resistogram. Computed tomography revealed nonunion of the femur one month later. Therefore revision surgery with autogenous bone grafting was performed. At the most recent follow-up, six months after rhBMP-2 use, there was still no complete bony bridging at the nonunion site.

## 4. Discussion

The off-label use of rhBMP-2 and rhBMP-7 in orthopedic surgery has considerably increased in the last years [20]. While rhBMP-2 application in adults has been evaluated in several clinical trials and case reports, its use in children and adolescents has been reported to a less extent. The scope of rhBMP-2 application in adults seems to be broad covering the use on almost every long bone, on the spine and in ENT surgery [12, 13, 21–23]. In children, few reports showed promising results after use of rhBMP-2 in pediatric spine fusion, for treatment of congenital pseudarthrosis of the tibia (CPT), and for maxillary reconstruction in cleft lip and palate patients [14, 16–19, 23].

Application of rhBMP-2 for the treatment of nonunion of the femur in pediatric patients was reported in only one study so far, focusing on complications associated with rhBMP-2 use at different sites in 81 children [15]. In 7 patients, rhBMP-2 was applied to the femur, but only 2 patients were treated for nonunion of the femur. In the other 5 cases, rhBMP-2 was used for the treatment of different disorders (after curettage and grafting of simple bone cyst, e.g.). It is not known whether union occurred, because the authors only report on the complications after rhBMP-2 use [15].

In the present study, rhBMP-2 combined with a locking plate has been used successfully to treat children and adolescents with nonunion of the femur. Union occurred in four of five patients at a mean of 12.1 months after rhBMP-2 use. After a mean follow-up of 62.5 months (17 to 100), union was still evident in all four patients and they were fully weight-bearing without pain. None of them had additional surgery for the treatment of femoral nonunion after rhBMP-2 use, except for removal of the locking plates at a mean of 16 months.

In their series of 19 children, Dohin et al. reported on the use of rhBMP-7 for the treatment of nonunion in different locations (e.g., congenital pseudarthrosis of the tibia, nonunion of the humerus, and sacral agenesis) including 3 children with nonunion of the femur [24]. Only one of these femoral nonunions healed after initial rhBMP-7 application; one healed after additional surgery with iliac crest bone grafting [24]. For the treatment of long bone nonunions, better results regarding the rate of union and the time to union have been reported for rhBMP-2 compared to rhBMP-7 use in a recently published study [25].

Two complications were thought possibly to be related to the use of rhBMP-2 in this case series. Postoperative swelling and overheating of the wound appeared in two patients after rhBMP-2 application, necessitating surgical treatment after 2 and 4.5 months, respectively, because of infection with* Staphylococcus aureus* in one case and spontaneous regression of these symptoms in the other case. Oetgen and Richards reported a complication rate of 17.5% after rhBMP-2 use at different sites in 81 children [15]. Three patients had a deep infection after surgery, and one patient with persistent nonunion of the femur developed a compartment syndrome 2 days after rhBMP-2 application, requiring fasciotomy and delayed closure [15].

The limitations of our study include its retrospective nature, the very small number of patients, and the lack of randomization. The strengths of our study are the long-term follow-up of 62.5 months (17 to 100) after rhBMP-2 use and the fact that all patients underwent nearly the same surgical procedure. A locking plate was placed in each case after resection of the pseudarthrosis and before rhBMP-2 was applied. Only one patient had short-term follow-up (6 months). But, in this case, deep infection after rhBMP-2 use occurred and the patient underwent two further operations with extensive debridement and removal of the locking plate. We believe that further follow-up does not bring any relevant information in this case due to the fact that the nonunion site was resected after rhBMP-2 application because of infection.

Based on the results of this retrospective study with a limited number of patients, the authors conclude that rhBMP-2 may provide a suitable therapeutic option to treat children and adolescents with nonunion of the femur, especially when operative treatment had failed before. However, prospective randomized controlled trials with more patients are warranted to investigate the long-term efficacy and safety of rhBMP-2 use in these cases.

## Figures and Tables

**Table 1:** Preoperative data.

Patients	Side	Age, years	Gender	Underlying disorder	Initial operation leading to rhBMP-2 treatment
1	Right	10, 3	Male	Infantile cerebral palsy with coxa antetorta	Femoral derotation osteotomy with use of a locking plate

2	Left	12, 0	Female	Congenital femoral deficiency with leg length inequality	Femoral derotation osteotomy and lengthening with use of Ilizarov device

3	Right	16, 2	Male	Infantile cerebral palsy with hip dislocation	Femoral shortening osteotomy with use of a locking plate combined with Pemberton osteotomy for open hip reduction

4	Right	11, 1	Female	Infantile cerebral palsy with coxa antetorta	Femoral derotation osteotomy with use of a locking plate

5	Right	5, 4	Male	Leg length inequality after septic coxitis with osteomyelitis of the proximal femur and previous femoral head reconstruction	Femoral lengthening with use of a vascularized fibular graft that was fixed with a K-wire

**Table 2:** Surgical procedure.

Patients	Surgical Procedure using rhBMP-2	rhBMP-2 dose (mg)
1	Removal of locking plate Resection of nonvascular tissue at the nonunion site Placing new locking plate	12

2	Removal of locking plate Resection of nonvascular tissue at the nonunion site Autogenous bone grafting from iliac crest Placing new locking plate	12

3	Removal of locking plate Resection of nonvascular tissue at the nonunion site Placing new locking plate	12

4	Removal of locking plate Resection of femoral pseudarthrosis Placing new locking plate	12

5	Resection of nonvascular tissue at the nonunion site between the fibular graft and proximal femur Autogenous bone grafting from iliac crest Placing a locking plate and external fixator	12

**Table 3:** Postoperative data.

Patients	Duration of follow-up (months)	Number of operations with use of rhBMP-2	Activities and brace at final follow-up	Radiographic end point
1	60	1	Active, no restrictions	Union

2	73	1	Active, no restrictions, shoe insert to compensate remaining leg length discrepancy	Union

3	6	1	Non-weight-bearing, verticalizer, knee-ankle-foot orthosis	Nonunion

4	17	1	Active, using crutches part-time, knee-ankle-foot orthosis	Union

5	100	1	Active, no restrictions	Union
